# Placental Morphologic Similarities Between ZIKV-Positive and HIV-Positive Pregnant Women

**DOI:** 10.3389/fimmu.2021.684194

**Published:** 2021-06-09

**Authors:** Daiane Cristine Martins Ronchi, Mineia Alessandra Scaranello Malaquias, Patrícia Zadorosnei Rebutini, Letícia Arianne Panini do Carmo, Plínio Cézar Neto, Emily Scaranello Marini, Amanda Prokopenko, Seigo Nagashima, Camila Zanluca, Claudia Nunes Duarte dos Santos, Lúcia de Noronha

**Affiliations:** ^1^ Laboratory of Experimental Pathology, Postgraduate Program of Health Sciences, School of Medicine, Pontifícia Universidade Católica do Paraná, Curitiba, Brazil; ^2^ Molecular Virology Laboratory, Instituto Carlos Chagas, Fundação Oswaldo Cruz, Curitiba, Brazil

**Keywords:** Zika virus, HIV, vertical transmission, placenta, morphometric analysis

## Abstract

Zika virus (ZIKV) caused global concern due to Brazil's unexpected epidemic, and it was associated with congenital microcephaly and other gestational intercurrences. The study aimed to analyze the placenta morphometric changes of ZIKV-infected pregnant women (ZIKV group; n = 23) compared to placentas of HIV-infected (HIV group; n = 24) and healthy pregnant women (N-control group; n = 22). It also analyzed the relationship between the morphometric results and pathological alterations on conventional microscopy, gestational trimester of infection, and presence of the congenital Zika syndrome (CZS). There was a significant increase in area (*p* = 0.0172), as well as a higher number of knots (*p* = 0.0027), sprouts (*p* < 0.0001), and CD163 +Hofbauer cells (HCs) (*p* < 0.0001) in the ZIKV group compared to the N-control group, suggesting that villous dysmaturity and HCs hyperplasia could be associated with ZIKV infections. The HIV group had a higher area (*p* < 0.0001), perimeter (*p* = 0.0001), sprouts (*p* < 0.0001), and CD163 + HCs (*p* < 0.0001) compared to the N-control group, demonstrating that the morphometric abnormalities found in the ZIKV and HIV group are probably similar. However, when ZIKV and HIV groups are compared, it was observed a higher number of sprouts (*p* = 0.0066) and CD163+ HCs (*p* < 0.0001) in the first one, suggesting that placental ZIKV congenital changes could be more pronounced.

## Introduction

During pregnancy, Zika virus (ZIKV) infection has been associated with fetal malformations, such as microcephaly, lissencephaly, cerebellar hypoplasia, hydrocephalus, polymicrogyria, abnormal development of the corpus callosum, and changes in neuronal migration and subcortical calcifications that configure the Congenital Zika Syndrome (1–9).

Recently, ZIKV caused global concern due to the unexpected epidemic of infection in Brazil, associated with congenital microcephaly and abortions, both of which have been more common when ZIKV infection occurred during the first trimester of gestation. Besides, severe cerebral malformations have not been described when the infection occurred in the third trimester, suggesting that the brain’s abnormal development associated with ZIKV could become the organogenesis period (10, 11).

CZS has been associated with placental alterations like an increase in the number of syncytial knots and sprouts, stromal disorders, villous immaturity, Hofbauer cells (HCs) hyperplasia, and vascular abnormalities (12, 13). The direct infection and replication of ZIKV in placenta tissues can be triggered by the infection of HCs (placental macrophage) in the chorionic villi (10). HCs appear to be the most frequently observed ZIKV-positive cells in the naturally infected human placentas and also may remain persistently infected until delivery. Even in the placenta samples with a short interval between the acute phase of infection and delivery time, ZIKV appears to be detected exclusively in HCs. Furthermore, villous immaturity may be related to congenital disorders caused by ZIKV infection, and it is also associated with an increase in HCs. The persistence of ZIKV-positive HCs in full-term placentas may indicate that these cells could provide a viral source for continued fetal infection and may be responsible for the transplacental transmission mediated by its migratory ability to reach the fetal vessels (12).

Human immunodeficiency virus (HIV) has also been associated with abortion, stillborn, preterm delivery, and other gestational intercurrences, but not with the congenital syndrome. However, the effects of HIV on the placentas remain poorly understood. The main target of HIV is CD4 T lymphocytes, but other cells expressing CD4 are also infected, like monocytes, macrophages, and dendritic cells, where HCs are included. Some of the alterations described include chorioamnionitis and deciduitis, and villitis, an increase in the number of syncytial knots and sprouts, stromal disorders like fibrin deposition and fibrosis, abnormalities of the villous maturation and infarction. Other authors have described placentas of HIV-infected pregnant with no pathological alterations on the conventional microscopy. On the other hand, morphometric techniques have usually shown alterations in villus diameter and perimeter, suggesting changes in villous maturation (14–18).

Given that, despite having different vertical transmission routes and outcomes, both HIV and ZIKV may produce similar morphological changes in placental tissues, such as villous immaturity and hyperplasia of HCs. Severe villitis, for example, does not appear to be a common form of placental injury in both cases. In addition, these two viruses can break through the placental barrier causing only subtle morphological alterations, resulting in placentas of the usual histological aspect under conventional microscopy (14–18).

Because of this, the present study aimed to analyze the placental morphometric changes in ZIKV-infected pregnant women and compare these changes with that found in HIV-infected pregnant women, considering gestational trimester of infection, presence of CZS, and pathological alterations on conventional microscopy as variables. In addition, this study also compares both groups (ZIKV and HIV) to the placentas of healthy (non-infected) pregnant women.

## Materials and Methods

### Ethical Approvals

The Brazilian National Ethics Committee approved the presented study under the number CAAE: 42481115.7.0000.5248. The authors confirm that all methods were carried out following relevant guidelines and regulations. Furthermore, the sample collection followed all relevant ethics and safety protocols. The data that support the findings of this study are available from the corresponding author upon reasonable request.

### Samples

The ZIKV-infected placenta group (ZIKV group) comprises 23 placentas that were formalin-fixed paraffin-embedded (FFPE) (12). The 23 patients gave birth to 15 term healthy and eight malformed babies, between 34 and 40 gestational weeks (average = 38; median = 38; SD = 2.17). All the 15 term healthy babies (37–40 gestational weeks) are alive. Of the eight malformed babies, five were preterm (34–36 gestational weeks). Still, regarding this group of malformed babies, four of them are alive, two had perinatal death and two were stillborn. The 23 mothers have at least two positive tests for ZIKV infection: anti-ZIKV IgM positive in the maternal blood and/or colostrum, positive RT-PCR in the maternal blood and/or urine, positive RT-PCR in the frozen placenta samples, positive RT-PCR and/or immunohistochemical test in the FFPE placenta samples. The newborn/stillborn additional samples were also positive: brain tissue RT-PCR and anti-ZIKV IgM in the blood (12).

The HIV-infected placenta group (HIV group) consisted of 24 FFPE placenta samples of HIV-positive pregnant women with no comorbidities. Pregnant women gave birth to healthy newborns between 33 and 40 weeks (average = 38.08; median = 38; SD = 1.99) of gestation in 2004 to 2005, when ZIKV was not circulating in Brazil. The placentas showed no pathological changes on the conventional microscopy. We did not observe villous maturation changes, and weights of the newborns were normal for gestational age (average = 2789.29 g; median = 2730 g; SD = 497.19 g; min-max = 1780–3890 g). Maternal age of this group ranged from 17 to 42 years (average = 26; median = 26; SD = 6.53). The placentas were from pregnant women who had been diagnosed with HIV before or during their pregnancy. The newborns were followed up until their HIV infection condition was defined as negative. All the babies are alive e HIV-seronegative. The viral loads and CD4/CD8 ratio were measured three to six times for most patients. The viral loads ranged from 13047.5 to 5760 (copies), and the CD4/CD8 ratio ranged from 0.65 to 0.35 during the 9 months of pregnancy. Antiretroviral therapy was administered at least 1 month before the birth in all patients (16).

The non-infected placenta group (N-control group) comprises 22 pregnant women that had prenatal without comorbidities. They gave birth to healthy newborns, between 34 and 40 gestational weeks (average = 38.19; median = 38; SD = 1.65), from 2004 to 2005, when ZIKV was not circulating in Brazil. The placentas did not present anatomopathological alterations. We did not observe villous maturation changes, and weights of the newborns were normal for gestational age (average = 2957.27 g; median = 2887.5 g; SD = 762.63 g; min–max = 1770–4410 g). Maternal age of this group ranged from 15 to 40 years (average = 26.06; median = 24; SD = 7.14). The pregnant woman and the newborn were followed up until discharge from the hospital (16).

The samples of three groups were matched by gestational age, which varied from 33 to 38 weeks. All the pregnant women were submitted to laboratory tests for congenital intrauterine infections (*TORCH = toxoplasmosis, rubella, cytomegalovirus, syphilis, and herpes*) with negative results. Analysis of gestational age showed no significant differences between the groups.

### Morphometric Analysis

Histological sections of all placentas were stained with hematoxylin & eosin (H&E) to evaluate the perimeter, diameter, and area of villi, the number of sprouts, syncytial knots, and villi numbers per medium power field (MPF). H&E sections were photographed at a magnification of 200× (MPF) using the Scanner Axion Scan.Z1, generating an average of 5,000 images. Unfocused, with artifacts, non-villous tissue representative (membranes, cord, decidua) images were excluded. The remaining images selected (about 1,000) had 100% of the field occupied with placental villi and were randomized to obtain about 100 images for each case of the three groups.

For all placentas, the perimeter, diameter, area of the villi, and basal membrane thickness were measured using Image-Pro Plus® 4 software, based on freehand drawing on 100 consecutive villi. After freehand villus’ contour, the program provided perimeter, diameters (major), area, and basal membrane thickness in micrometers or square micrometers (µm/µm^2^) (16).

To evaluate the syncytial knots and sprouts per villi, the same 100 MPF/H&E images were used and submitted to simple counting of these microscopic structures (12).

### Immunohistochemical Analysis

Histological sections of the placentas were fixed on electrically charged glass slides and subsequently dewaxed with heated xylol (37°C), dehydrated with successive baths of absolute ethyl alcohol, and rehydrated with water. Methyl alcohol and hydrogen peroxide were used to block endogenous peroxidase and distilled water and hydrogen peroxide for the second block. They were incubated with anti-CD163 primary antibody (type: polyclonal/rabbit; clone/code: 14215; dilution: 1:1000; source: Thermo Fisher) for 1 h and with secondary antibody associated with the dextran polymer (Spring Bioscience, Pleasanton, USA) for 30 min. DAB/substrate complex (DAB, DakoCytomation) was added onto the slides, followed by counterstaining with Mayer’s hematoxylin, dehydration with ethyl alcohol baths, clarification with xylol, and blending with Canada balsam (12).

The 30 HPF (high power field = 400×) were analyzed by counting the number of villi and CD163+ HCs per villi in all three study groups.

The images were obtained from random sample regions without the interference of an observer. The morphometric measurements and the score of CD163 positive cells were performed blindly.

### Statistical Analyses

The results were described by means, standard deviations, medians, minimum, and maximum values. The comparison of the groups concerning quantitative variables was performed using the non-parametric Kruskal-Wallis test or t-test. The Shapiro-Wilk test evaluated the normality condition. Values of *p* < 0.05 indicated statistical significance. The data were analyzed using the IBM SPSS Statistics v.20.0 software. Armonk, NY, USA: IBM Corp.

## Results

### Morphometric Alterations of the HIV and ZIKV Groups

**Figure 1:**
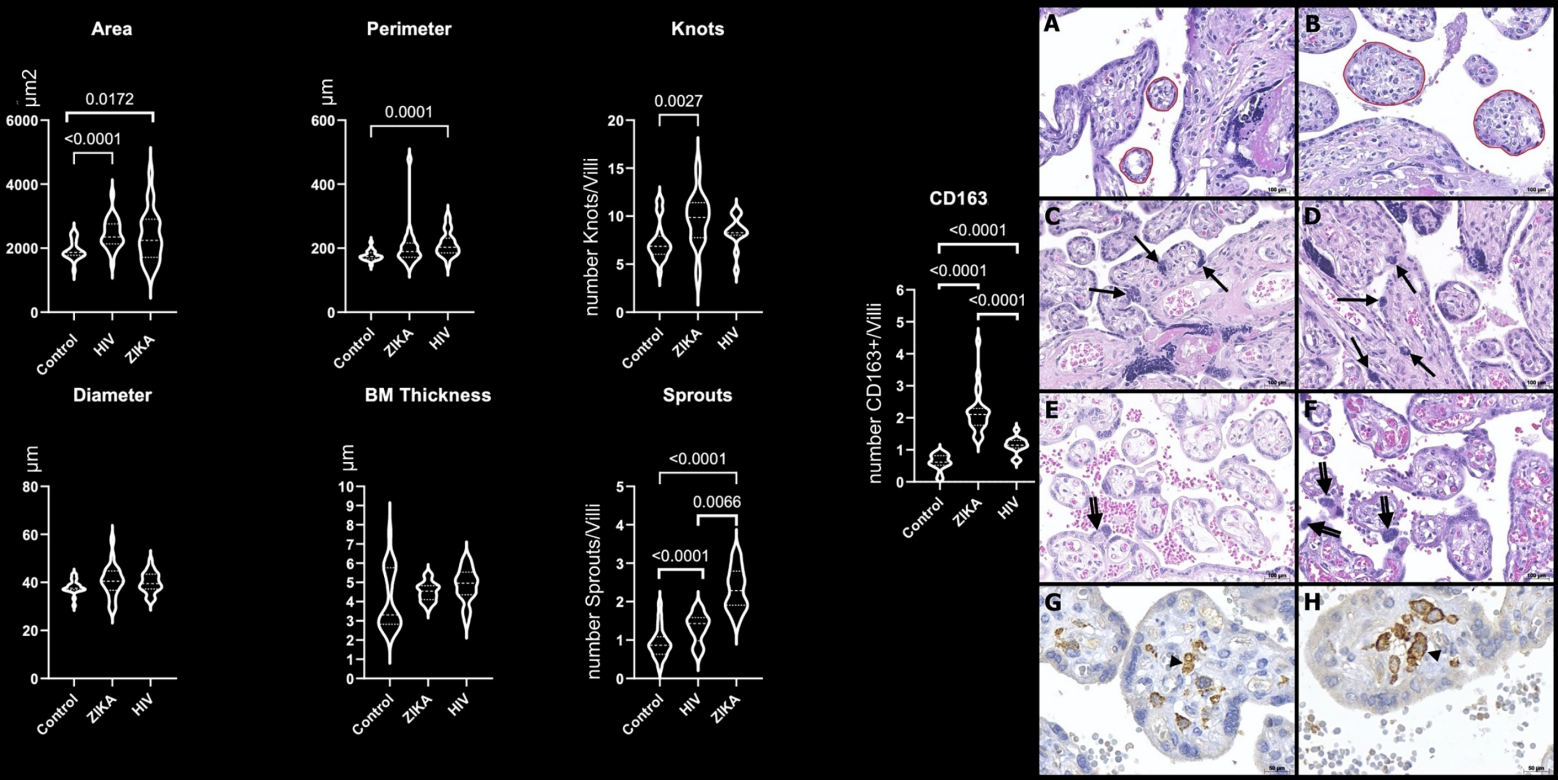
Morphometric analysis of placental specimens from women infected with ZIKV during the pregnancy compared to the HIV and N-control groups. Perimeter, diameter, and basal membrane (BM) thickness in µm; area in µm^2^; number of knots, sprouts, and CD163+ HCs per villi (CD163/villi). Photomicrography of a placental sample stained with H&E showing the perimeter of villi (red freehand drawing) in N-control group **(A)** and ZIKV group **(B)**; the number of syncytial knots/villi (arrows) in N-control group **(C)** and ZIKV-group **(D)**; the number of sprouts/villi (double arrows) in N-control group **(E)** and ZIKV group **(F)**. Original magnification: 200×. Scale bars: 100 μm. Photomicrography of immunostaining with CD163 highlighting Hofbauer cell (arrowhead) in the N-control group **(G)** and ZIKV group **(H)**. Original magnification: 400×. Scale bars: 50 μm.

The analysis of the area (*p* = 0.0172) and the number of knots (*p* = 0.0027), sprouts (*p* < 0.0001), and CD163+ HCs (*p* < 0.0001) in the ZIKV group demonstrated larger immature chorionic villi with a higher number of knots and sprouts and HCs hyperplasia when compared with the N-control group (**Figure 1** and **Supplementary Figure 1**). 

HIV group placentas with no pathological alterations on conventional microscopy also showed changes in villous maturation and HC hyperplasia by morphometry analysis compared to the N-control group. The area (*p* < 0.0001), perimeter (p = 0.0001), number sprouts (*p* < 0.0001), and CD163+ HCs (*p* < 0.0001) of HIV group were higher than the N-control group (**Figure 1** and **Supplementary Figure 1**).

The ZIKV group placentas showed higher values of the number of sprouts (*p* < 0.0066) and CD163+ HCs (*p* < 0.0001) compared to the HIV group (**Figure 1** and **Supplementary Figure 1**).

### Trimester of ZIKV Infection

Morphometric analyses were performed in placental samples from mothers who were infected with ZIKV during the first (n = 4), second (n = 8), and third trimesters of pregnancy (n = 6). In five placenta samples, the trimester of infection was unknown. The perimeter (*p* = 0.0292), number of knots (*p* = 0.0062), sprouts (*p* < 0.0001), and CD163+ HCs (*p* < 0.0001) showed significant differences by the trimester of infection. The most relevant differences were observed between the second trimester and third trimester of infection versus the N-control group, revealing second-/third-trimester ZIKV placentas with villous dysmaturity and HCs hyperplasia compared to the control placentas (**Table 1**).

**Table 1:** Median (max-min) and *p*-value of morphometric data in the gestational trimester of infection, presence of placenta pathological alterations, and CZS.

		Gestational Trimester of Infection^†^			Pathological Alterations of Placenta on Conventional Microscopy§		Congenital Zika Syndrome*	
Variables	N-CONTROL	First (n = 4)	Second (n = 8)	Third (n = 6)	No (n = 15)	Yes (n = 8)	No (n = 15)	Yes (n = 8)
**Diameter (µm)**	37.6 (43.4–30.4)	38.3 (46.3–29.1)	40.2 (45.3–32.1)	39.8 (44.6–29.1)	40.1 (50.6–29.1)	44.9 (57.7–36.6)	40.0 (45.3–29.1)	45.5 (57.7–36.6)
		NS			**0.0226**		**0.0109**	
**Area (µm^2^)**	1867 (2496–1314)	1812.9 (3536.9–1228.8)	2119.4 (2927.2–1377.9)	2241.8 (2917.7–1527.3)	2165.1(3671.2–1228.8)	2907.4 (4354.4–1673.3)	2119.4 (2927.2–1228.8)	2897.1 (4354.4–1673.3)
		NS			NS		**0.0102**	
**Perimeter (µm)**	174 (219–148)	174.2 (237.2–142.2)	182.1 (477.7–163.2)	186.2 (203.8–154.3)	181.2 (477.7–142.2)	208.9 (257.6–163.2)	180.3 (477.7–142.2)	214.0 (257.6–176.9)
		**0.0292****^a^**			**0.0212**		**0.0035**	
**Number of knots/villi**	6.9 (11.7–4.2)	9.9 (11.6–7.9)	11.1 (14.3–9.3)	8.6 (15.5–4.7)	9.8 (15.5–4.7)	10.2 (11.6–3.5)	10.1 (15.5–4.7)	9.8 (11.6–3.5)
		**0.0062****^a^**			**0.0101**		**0.0054**	
**Number of sprouts/villi**	0.9 (1.9–0.3)	1.9 (3.2–1.6)	2.4 (3.1–1.4)	2.1 (2.6–1.8)	2.4 (3.2–1.6)	2.0 (3.3–1.4)	2.2 (3.2–1.4)	2.8 (3.3–1.7)
		**<0.0001****^b^**			**<0.0001**		**<0.0001**	
**Number of cells CD163+/villi**	0.6 (1.0–0.1)	2.7 (3.3–2.0)	2.2 (2.3–1.7)	1.9 (2.3–1.7)	2.0 (2.4–1.3)	2.5 (4.4–1.7)	2.0 (2.4–1.3)	2.8 (4.4–1.4)
		**<0.0001****^c^**			**<0.0001**		**<0.0001**	

Analysis only for the ZIKV group.

^†^Five cases of unknown gestational trimester.

^§^Main pathological findings: umbilical artery agenesis (1), mild acute funisitis (1), and villous immaturity (6).

*Major central nervous malformations: microcephaly (3), spina bifida (1), hydrocephalus (2), and encephalocele (1).

a p value refers to the second trimester vs. N-control.

b p-value refers to the e third trimester vs. N-control and first vs. the third trimester.

c p-value refers to the third trimester vs. N-control; first vs. the second and third trimester. Kruskal-Wallis test and t-test; values of p < 0.05 indicated statistical significance.

NS, not significant p-value. Bold values = Statistically significant values.

### Pathological Alterations

ZIKV group placentas with and without pathological alterations by conventional microscopy were compared. It was observed that placentas with pathological changes presented higher diameter (*p* = 0.0226), perimeter (*p* = 0.0212), number of knots (*p* = 0.0101), number of sprouts (*p* < 0.0001), and CD163+ HCs (*p* < 0.0001) compared to placentas without pathological alterations (**Table 1**). However, placentas considered within normal standards also presented morphometric changes characterized by higher area, perimeter, number of knots, sprouts, and CD163+ HCs compared to the N-control group (*p* < 0.05).

### Congenital Zika Syndrome

The status of the newborns (with or without CZS) was also analyzed. The diameter (*p* = 0.0109), area (*p* = 0.0102), perimeter (*p* = 0.0035), number of knot (*p* = 0.0054), number of sprouts (*p* < 0.0001), and CD163+ HCs (*p* < 0.0001) were higher in placentas of newborns with CZS compared with placentas of newborns without this condition (**Table 1**).

## Discussion

### Morphometric Alterations of HIV and ZIKV Groups

The findings showed significant enlargement of the area of the ZIKV group when compared with the N-control group. A higher number of knots, sprouts, and CD163+ HCs were also noticed.

Syncytial knots are syncytiotrophoblasts’ specializations, and their severe increase in late gestation indicates early maturation (12). Syncytial sprouts are markers of trophoblast proliferation; they are seen frequently during early pregnancy and are increased in the villous dysmaturity (19, 20).

HCs, the most frequently ZIKV-positive cells, are placental villous macrophages of fetal origin, and alterations in their numbers (hyperplasia) and biological features are associated with complications in pregnancy. HCs play a role in diverse functions, such as placental vasculogenesis, immune regulation, and the secretion of enzymes and cytokines across the maternal-fetal barrier. In addition, there is some evidence suggesting the involvement of HCs in the development of placental villi (12).

This study’s findings corroborate with studies that showed a delay in villous maturation and signs of the HCs hyperplasia in ZIKV-infected placentas. These alterations could damage the chorionic villi, such as calcification, necrosis, Wharton jelly sclerosis, fibrin deposition, and a significant villi size increase (11, 21–23). We could conclude that all of the anatomopathological parameters could be confirmed by the morphometric data and may be used to describe ZIKV-infected placentas.

Other findings showed that the HIV group had a larger area, perimeter, number of sprouts, and CD163+ HCs compared to the N-control group. Studies also revealed that placentas exposed to HIV infection exhibited the following microscopic features: edema, villous immaturity, focal necrosis of trophoblasts, numerous HCs, intervillous fibrin deposition, and chorangiosis (17, 18, 24). However, when those patterns are subtle or minimal, pathologists cannot make the diagnosis. Given that, morphometric techniques may be helpful to identify subtle abnormalities.

Rabelo et al. (22) showed ZIKV NS1 protein in the decidual and endothelial cells of the maternal decidua and CTB, STB, and HCs in the third trimester placental tissues associated with an HIV-exposed, but uninfected, infant with severe congenital Zika syndrome. Nonetheless, the maternal HIV infection could have contributed to the permissiveness of other placental cell types to ZIKV infection.

Finally, when both groups (ZIKV and HIV) were compared, no statistically significant results were found, except for the number of sprouts and CD163+ HCs higher in the ZIKV group. Thus, it seems that HCs hyperplasia and sprouting/dysmaturity villi may be more pronounced and characteristic in the ZIKV-infected placentas (12, 17). Even though placental changes, such as dysmaturity and hyperplasia of HCs, can be seen in other maternal-fetal diseases, such as congenital infections (TORCH) and diabetes, in the absence of these comorbidities, this aspect may help the pediatric pathologists to suspect the diagnosis of ZIKV vertical transmission. This study also demonstrates that the morphometrical abnormalities finding in ZIKV and HIV groups are very similar, despite having different vertical transmission routes and outcomes since ZIKV is a teratogenic virus and HIV is not. In addition, vertical HIV transmission is much rarer than that of ZIKV, but it can increase perinatal and intrauterine deaths (**Supplementary Figure 1**) (14, 17, 18).

### Trimester of ZIKV Infection

When the gestational trimester of infection was analyzed, it was observed that most of the differences between the ZIKV and N-control groups appear to be when infection occurred in the second or third trimester. Since all newborns of this study were in the third trimester (term or preterm), the shorter time that elapsed between the moment of ZIKV infection and the birth may be an explanation for more pronounced changes in these placentas.

The number of HCs also showed differences between groups, suggesting that these cells may have early hyperplasia, and this hyperplasia seems to be maintained throughout the gestational period, although it decreases in intensity over the months. This fact appears to agree with the hypothesis that these cells can work as a reservoir of ZIKV (12, 25).

Regardless of the trimester in which the infection occurred, as ZIKV is detected in placental cells until the end of pregnancy, it is plausible to speculate that the infection of the fetus could happen as a secondary event. In some cases, those abnormalities are only detected months after the delivery (20, 26–29).

### Pathological Alterations

Fifteen of 23 ZIKV group placentas were diagnosed without pathological alterations for the pediatric pathologist. However, eight of them had pathological alterations on conventional microscopy, mainly villous immaturity. When placentas with and without pathological alterations were compared, placentas diagnosed with villous immaturity had a higher diameter, perimeter, number of knots, and CD163+ cells. This means that pathologists probably identified those alterations on conventional microscopy and, altogether, termed villous immaturity, so they did not need morphometry techniques to make these diagnoses.

On the other hand, ZIKV-group placentas with no pathological alterations also showed higher diameter, perimeter, and number of knots, sprouts, and CD163+ cells to the N-control group. Therefore, conventional microscopy cannot identify subtle alterations that morphometry could find.

### Congenital Zika Syndrome

This study showed significant enlargement of the villi’s diameter, area, and perimeter and the number of sprouts and CD163+ HCs in the group that had CZS. In addition, eight infants had fetal malformations related to ZIKV infection during pregnancy. However, 15 women had the onset of ZIKV symptoms during pregnancy and gave birth to infants without CZS.

This study observed that ZIKV causes essential alterations in the placenta’s villous, leading to congenital disorders, stillborn, and neonatal death. We could also conclude that morphometric parameters may be biomarkers for CZS since they are more pronounced in malformed newborns. These data could help in the clinical follow-up of newborns with subclinical congenital disorders or even unapparent at birth.

In conclusion, there are placental dysmaturity alterations after ZIKV infection during pregnancy. Very similar placental alterations could be demonstrated on the HIV-infected pregnant women, but sprouting and HCs hyperplasia may be less pronounced in this group. Also, the morphometric analysis revealed villous dysmaturity even in placentas diagnosed within the usual standards by the routine exams. The second and third gestational trimester infections generated more villous dysmaturity and HCs hyperplasia than those pregnant women who became infected in the first trimester. In addition, placentas whose babies had CZS showed more pronounced changes than those without CZS. These alterations may help understand the aspect of ZIKV infection related to placental damage and congenital disabilities and possible deficiencies that might appear after birth.

## Data Availability Statement

The raw data supporting the conclusions of this article will be made available by the authors, without undue reservation.

## Ethics Statement

The studies involving human participants were reviewed and approved by the ethics committee of the Oswaldo Cruz Foundation (Fiocruz) Brazilian National Ethics Committee of Human Experimentation. The patients/participants provided their written informed consent to participate in this study.

## Author Contributions

LN, CZ, and CNDS contributed to the study design. CZ performed the experiments. SN prepared the materials for analyses. DCMR, PZR, LAPC, PCN, and AP performed the morphometric analyses. PZR participated in the patient follow-up. MASM and ESM participated in sample identification/distribution. DCMR, MASM, PZR, and LN analyzed the results and wrote the manuscript. All authors contributed to the article and approved the submitted version.

## Funding

This research was funded by Fiocruz, CNPq (439968/2016-0), CAPES Zika Fast Track 481-2016 (88887.116626/2016-01), and Fundação Araucária (CP04/2016). CNDS and LN have a CNPq fellowship.

## Conflict of Interest

The authors declare that the research was conducted in the absence of any commercial or financial relationships that could be construed as a potential conflict of interest.

## Supplementary Material

Supplementary Figure 1 Illustrative figure shows the morphometric differences between N-control and ZIKV group and between N-control and HIV group, respectively. However, it also represents similar morphometrics results when the ZIKV group was compared with the HIV group. The N-control group’s syncytial membranes are more efficient in exchanges because the vessels are closer to the cytotrophoblast. In ZIKV- and HIV-infected placentas, villus increases in size, moving the vessel away from the trophoblast layer, distancing the syncytiotrophoblast membranes cause intrauterine death, premature, low birth weight babies, and/or other malformations. Created with www.biorender.com.

## References

[1] Neu N Duchon J Zachariah P. TORCH Infections. Clin Perinatol (2015) 42(1):77–103(viii). 10.1016/j.clp.2014.11.001 25677998

[2] Noronha Ld Zanluca C Azevedo ML Luz KG Santos CN. Zika Virus Damages the Human Placental Barrier and Presents Marked Fetal Neurotropism. Mem Inst Oswaldo Cruz (2016) 111(5):287–93. 10.1590/0074-02760160085 PMC4878297 27143490

[3] Ayres CFJ. Identification of Zika Virus Vectors and Implications for Control. Lancet Infect Dis (2016) 16(3):278–9. 10.1016/S1473-3099 26852727

[4] Solomon IH Milner DA Folkerth RD. Neuropathology of Zika Virus Infection. J Neuroinfect Dis (2016) 7(2):220. 10.4172/2314-7326.1000220 27525286 PMC4982465

[5] Hughes BW Addanki KC Sriskanda AN McLean E Bagasra O. Infectivity of Immature Neurons to Zika Vírus: A Link to Congenital Zika Syndrome. EBioMedicine (2016) 10):65–70. 10.1016/j.ebiom.2016.06.026 27364784 PMC5006602

[6] Oehler E Watrin L Larre P Leparc-Goffart I Lastère S Valour F. ZIKV Infection Complicated by Guillain-Barré Syndrome: Case Report, French Polinesia, December 2013. Euro Surveill (2014) 19(9):1–3. 10.2807/1560-7917.es2014.19.9.20720 24626205

[7] Brasil P Pereira JP Moreira ME Nogueira RMR Damasceno L Wakimoto M. Zika Virus Infection in Pregnant Women in Rio De Janeiro. N Engl J Med (2016) 375:2321–34. 10.1056/NEJMoa1602412 PMC5323261 26943629

[8] Melo AS Aguiar RS Amorim MMR Arruda MB Melo FO Ribeiro STC. Congenital Zika Virus Infection: Beyond Neonatal Microcephaly. JAMA Neurol (2016) 73:1407–16. 10.1001/jamaneurol.2016.3720 27695855

[9] Schuler-Faccini L Ribeiro EM Feitosa IM Horovitz DDG Cavalcanti DP Pessoa A. Brazilian Medical Genetics Society–Zika Embryopathy Task Forceet. Possible Association Between Zika Virus Infection and Microcephaly–Brazil, 2015. Morb. Mortal Wkly Rep (2016) 65:59–62. 10.15585/mmwr.mm6503e2 26820244

[10] Petersen LR Jamieson DJ Powers AM Honein MA. Zika Virus. Baden LR, Editor. N Engl J Med (2016) 374(16):1552–63. 10.1056/NEJMra1602113 27028561

[11] Bhatnagar J Rabeneck DB Martines RB Reagan-Steiner S Ermias Y Estetter LBC. Zika Vírus RNA Replication and Persistence in Brain and Placental Tissue. Emerging Infect Dis (2017) 23(3):405–14. 10.3201/eid2303.161499 PMC5382738 27959260

[12] Noronha L Zanluca C Burger M Suzukawa AA Azevedo M Rebutini PZ. Zika Virus Infection At Different Pregnancy Stages: Anatomopathological Findings, Target Cells and Viral Persistence in Placental Tissues. Front Microbiol (2018) 9:2266. 10.3389/fmicb.2018.02266 30337910 PMC6180237

[13] Souza AS Dias CM Braga FDCB Terzian ACB Estofolete CF Oliani AH. Fetal Infection by Zika Virus in the Third Trimester: Report of 2 Cases. Clin Infect Dis (2016) 63:1622–5. 10.1093/cid/ciw613 27601223

[14] Maartens G Celum C Lewin SR. HIV Infection: Epidemiology, Pathogenesis, Treatment, and Prevention. Lancet (2014) 384(9939):258–71. 10.1016/S0140-6736(14)60164-1 24907868

[15] Yang SW Cho EH Choi SY Lee YK Park JH Kim MK. Dc-SIGN Expression in Hofbauer Cells may Play an Important Role in Immune Tolerance in Fetal Chorionic Villi During the Development of Preeclampsia. J Reprod Immunol (2017) 124:30–7. 10.1016/j.jri.2017.09.012 29049918

[16] Baurakiades E Martins APC Moreschi V Souza CDA Abujamra K Saito AO. Histomorphometric and Immunohistochemical Analysis of Infectious Agents, T-cell Subpopulations and Inflammatory Adhesion Molecules in Placentas From HIV-seropositive Pregnant Women. Diagn Pathol (2011) 6:101. 10.1186/1746-1596-6-101 22024147 PMC3214179

[17] López CL Pires ARC Fonseca EC Rodrigues FR Braga Neto AR Herdy GVH. Anatomopathological Characterization of Placentas From HIV+ Patients Associated With p24 Expression. J Bras Patol Med Lab (2013) 49(6):437–45. 10.1590/S1676-24442013000600010

[18] Obimbo MM Zhou Y McMaster MT Cohen CR Qureshi Z Ong’ech J. Placental Structure in Preterm Birth Among HIV-positive Versus HIV-negative Women in Kenya. J Acquir Immune Defic Syndr (2019) 80(1):94–102. 10.1097/QAI.000000000000187 30272633 PMC6289800

[19] Loukeris K Sela R Baergen RN. Syncytial Knots as a Reflection of Placental Maturity: Reference Values for 20 to 40 Weeks Gestational Age. Pediatr Dev Pathol (2010) 13:305–9. 10.2350/09-08-0692-OA.1 20017638

[20] Aagaard KM Lahon A Suter MA Arya RP Seferovic MD Vogt MB. Primary Human Placental Trophoblasts are Permissive for Zika Virus (ZIKV) Replication. Sci Rep (2017) 7:41389. 10.1038/srep41389 28128342 PMC5269613

[21] Tabata T Petitt M Puerta-Guardo H Michlmayr D Wang C Fang-Hoover J. Zika Virus Targets Different Primary Human Placental Cells, Suggesting Two Routes for Vertical Transmission. Cell Host Microbe (2016) 20(2):155–66. 10.1016/j.chom.2016.07.002 PMC5257282 27443522

[22] Rabelo K Fernandes RCSC Souza LJ Souza TL Santos FB Nunes PCG. Placental Histopathology and Clinical Presentation of Severe Congenital Zika Syndrome in a Human Immunodeficiency Virus-Exposed Uninfected Infant. Front Immunol (2017) 8:1704. 10.3389/fimmu.2017.01704 29270171 PMC5725436

[23] Zare Mehrjardi M Shobeirian F. The Role of the Placenta in Prenatally Acquired Zika Virus Infection. Virus Dis (2017) 28(3):247–9. 10.1007/s13337-017-0399-z PMC5684994 29291210

[24] Anderson VM. The Placental Barrier to Maternal HIV Infection. Obstet Gynecol Clin North Am (1997) 24(4):797–820. 10.1016/s0889-8545(05)70345-4 9430168

[25] Zanluca C De Melo VCA Mosimann ALP dos Santos GIV dos Santos CND Luz K. First Report of Autochthonous Transmission of Zika Virus in Brazil. Mem Inst Oswaldo Cruz (2015) 110(4):569–72. 10.1590/0074-02760150192 PMC4501423 26061233

[26] Aragão M Holanda A Brainer-Lima A Petribu NCL Castillo M van der Linden V. Nonmicrocephalic Infants With Congenital Zika Syndrome Suspected Only After Neuroimaging Evaluation Compared With Those With Microcephaly At Birth and Postnatally: How Large is the Zika Virus “Iceberg”? AJNR Am J Neuroradiol (2017) 38:1427–34. 10.3174/ajnr.A5216 PMC7959892 28522665

[27] Ventura L Ventura C Lawrence L van der Linden V van der Linden A Gois AL. Visual Impairment in Children With Congenital Zika Syndrome. J AAPOS (2017) 21:295–9. 10.1016/j.jaapos.2017.04.003 28450178

[28] Luo H Winkelmann ER Fernandez-Salas I Li L Mayer SV Danis-Lozano R. Zika, Dengue and Yellow Fever Viruses Induce Differential Anti-Viral Immune Responses in Human Monocytic and First Trimester Trophoblast Cells. Antiviral Res (2018) 151:55–62. 10.1016/j.antiviral.2018.01.003 29331320 PMC5844857

[29] Sheridan MA Yunusov D Balaraman V Alexenko AP Yabe S Verjovski-Almeida S. Vulnerability of Primitive Human Placental Trophoblast to Zika Virus. Proc Natl Acad Sci (2017) 114(9):E1587–96. 10.1073/pnas.1616097114 PMC5338554 28193876

