# Supplementary figures and images for: Placental Morphologic Similarities Between ZIKV-Positive and HIV-Positive Pregnant Women
Source: Front Immunol. 2021 Jun 9;12:684194. doi: 10.3389/fimmu.2021.684194 (PMC8219962; doi:10.3389/fimmu.2021.684194)

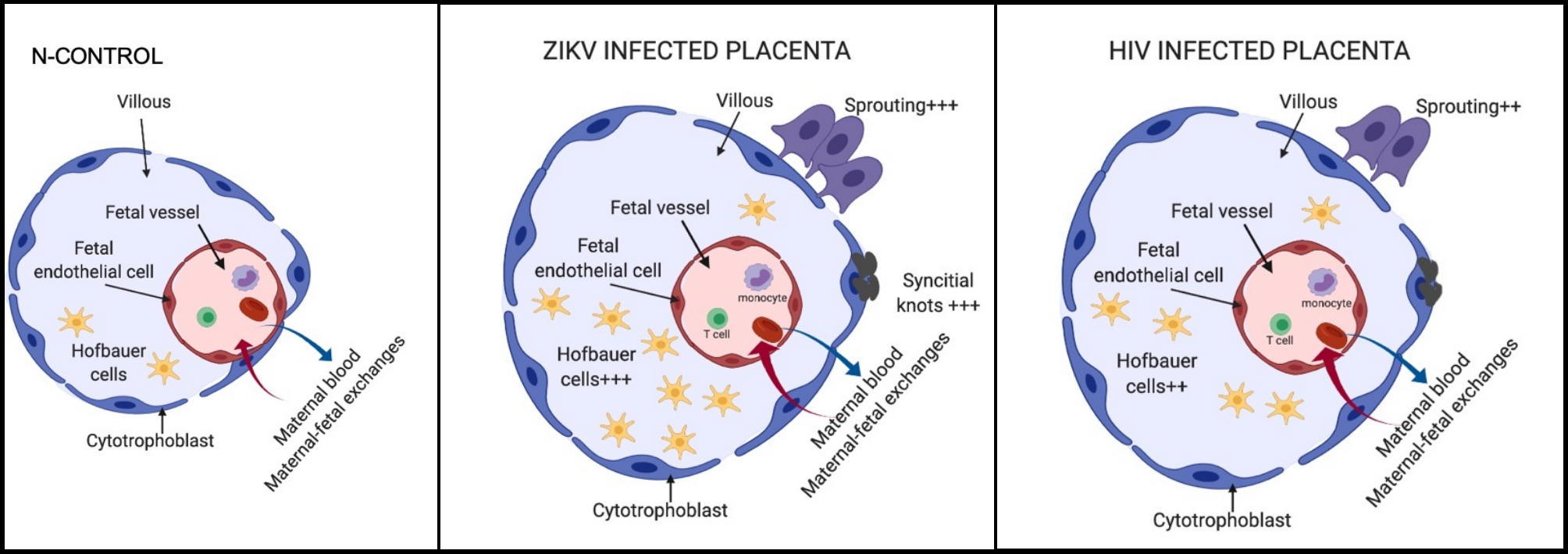

Supplement: Supplementary Figure 1 — Illustrative figure shows the morphometric differences between N-control and ZIKV group and between N-control and HIV group, respectively. However, it also represents similar morphometrics results when the ZIKV group was compared with the HIV group. The N-control group’s syncytial membranes are more efficient in exchanges because the vessels are closer to the cytotrophoblast. In ZIKV- and HIV-infected placentas, villus increases in size, moving the vessel away from the trophoblast layer, distancing the syncytiotrophoblast membranes cause intrauterine death, premature, low birth weight babies, and/or other malformations. Created with www.biorender.com. [file Image_1.tiff]
